# Generation and molecular characteristics of a highly attenuated GPV strain through adaptation in GEF cells

**DOI:** 10.1186/s12917-020-02673-0

**Published:** 2020-11-23

**Authors:** Hongxia Shao, Yuchen Jiang, Huisha Yuan, Lifei Ji, Wenjie Jin, Kun Qian, Jianqiang Ye, Aijian Qin

**Affiliations:** 1 Key Laboratory of Jiangsu Preventive Veterinary Medicine, Key Laboratory for Avian Preventive Medicine, Ministry of Education, College of Veterinary Medicine, Yangzhou University, No.12 East Wenhui Road, Yangzhou, 225009 Jiangsu China; 2 Jiangsu Co-innovation Center for Prevention and Control of Important Animal Infectious Diseases and Zoonoses, Yangzhou, 225009 Jiangsu China; 3 Joint International Research Laboratory of Agriculture and Agri-Product Safety, the Ministry of Education of China, Yangzhou University, Yangzhou, 225009 Jiangsu China; 4 Institutes of Agricultural Science and Technology Development, Yangzhou University, Yangzhou, 225009 Jiangsu China

**Keywords:** Goose parvovirus, Attenuated virus, Gene mutation, Vaccine, Virulence

## Abstract

**Background:**

Goose parvoviruses (GPVs) spread globally and cause a huge economic loss to the poultry industry. Although the attenuated GPV vaccines play a key role in preventing the disease caused by GPV, the molecular basis for the attenuation of GPV is barely known.

**Results:**

A highly attenuated GPV strain, GPV-CZM-142, was generated through blindly passaging of the highly pathogenic strain, GPV-CZM, in goose embryonic fibroblasts (GEF) for 142 generations. The GEF-adapted GPV strain’s virulence was 10,000 times weaker than its wild type counterpart, GPV-CZM, based on the ELD_50_ (50% Embryo Lethal Dose). By comparing with the wild type strain, genome sequencing analysis identified adapted mutations either in ITR or in NS and VP1 of GPV-CZM-142.

**Conclusions:**

The highly attenuated GPV strain, GPV-CZM-142, provides a GPV vaccine candidate, and the identified virulence-related mutations give a novel insight into the molecular determinants of GPV virulence.

## Background

Goose parvovirus (GPV) belongs to the genus *parvoviruses*, family *Parvoviridae*. GPV is a non-enveloped and single-stranded DNA virus. The genome of GPV contains two inverted terminal repeats (ITRs) and two major open reading frames (ORFs). ITR carries important elements for viral replication and encapsidation. The two major ORFs encode the non-structural protein (NS) and the capsid proteins (VP1/2/3). VP1 is the largest capsid protein, which shares a common region of C-terminus with VP2 and VP3. As a globally spread virus, GPV was first isolated and identified in 1956 by Fang in China [1, 2]. Its infection mainly causes acute contagious and septic diseases in goslings and Muscovy ducks with age younger than 30 d old [3]. The intestinal suppository is a characteristic lesion for GPV infection in goslings [4]. Although the attenuated GPV vaccine and its yolk antibody play a key role in preventing disease deterioration caused by GPV, GPV is still frequently isolated in the GPV-vaccinated goose. Also, the current strategies for GPV prevention and control are challenged with the following problems: later onset of GPV infection in goose/duck individuals, more emergence of the GPV mutants, and the co-infection of GPV with other pathogens (e.g., goose astrovirus). However, the molecular basis for virulence of GPV remains unclear. In this study, a highly pathogenic GPV strain, GPV-CZM, was continuously passaged in the GEF for 142 generations to generate a highly attenuated GPV strain, GPV-CZM-142, followed by identification mutations responsible for the attenuation by genomic sequencing analysis.

## Results

### Highly attenuated GPV-CZM-142 generated through adaptation to GEF

To generate an attenuated GPV vaccine candidate, a highly pathogenic GPV strain GPV-CZM was continuously passaged in GEFs underwent a total of 142 generations. To evaluate the pathogenicity of the adapted GPV-CZM, the ELD_50_ of wild type GPV-CZM, the 70th generation of GPV-CZM-70, and the 142nd generation of GPV-CZM-142 were tested in goose embryos. The goose embryos’ mortalities infected with the wild type GPV-CZM were 100, 100, 80, 60, and 40% at the infection dose of 1:10, 1: 10^2^, 1: 10^3^, 1: 10^4^, and 1: 10^5^ dilutions, respectively. In contrast, those of the goose embryos infected with GPV-CZM-70 were 20, 0, 0, 0 and 0%, respectively. Notably, GPV-CZM-142 was not lethal to goose embryos at these doses. Based on the viral TCID_50_ (50% Tissue Culture Infectious Dose) titers of these GPV strains, The ELD_50_ of GPD-CZM, GPV-CZM-70, and GPV-CZM-142 were 1.46 TCID_50_, 10^5^ TCID_50,_ and > 10^5^ TCID_50_, respectively (Table 1). These data demonstrate that the GPV-CZM-70 and GPV-CZM-142 strains are highly attenuated compared with the wild type GPV-CZM.

**Table 1:** Detection of the ELD_50_ of GPV-CZM-70 and GPV-CZM-142

Virus name	Virus titer		
	TCID_50_/ml	ELD_50_/ml	TCID_50_/ELD_50_
GPV-CZM	1.58 × 10^5^	1.08 × 10^5^	1.46
GPV-CZM-70	5 × 10^5^	5	10^5^
GPV-CZM-142	5 × 10^5^	< 5	> 10^5^

### Sketch on the genome sequences of different GPV strains

**Fig. 1:**
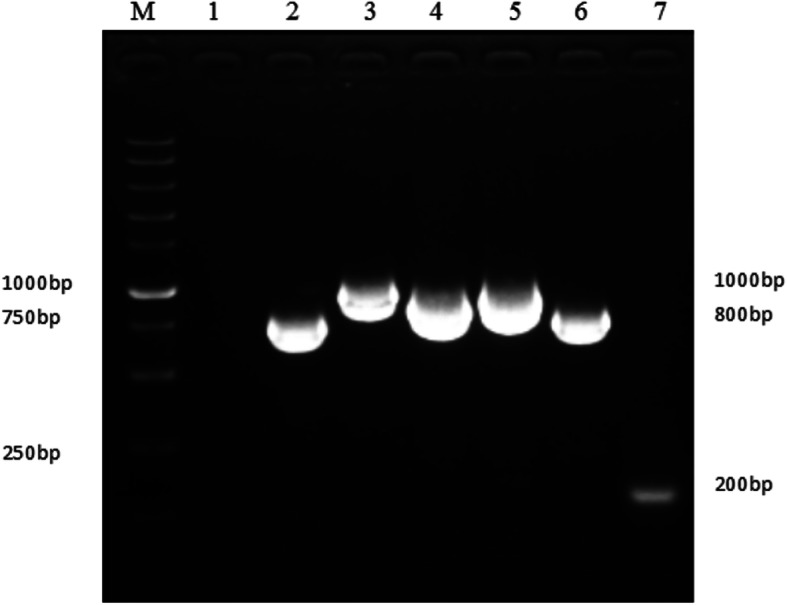
PCR amplification for the genome of GPV. Lane M. 1 kb Marker; Lane 1. Negative control; Lane 2–7. PCR fragments using primers F2/R2, F3/R3, F4/R4, F5/R5, F6/R6, and F1/R1, respectively

**Fig. 2:**
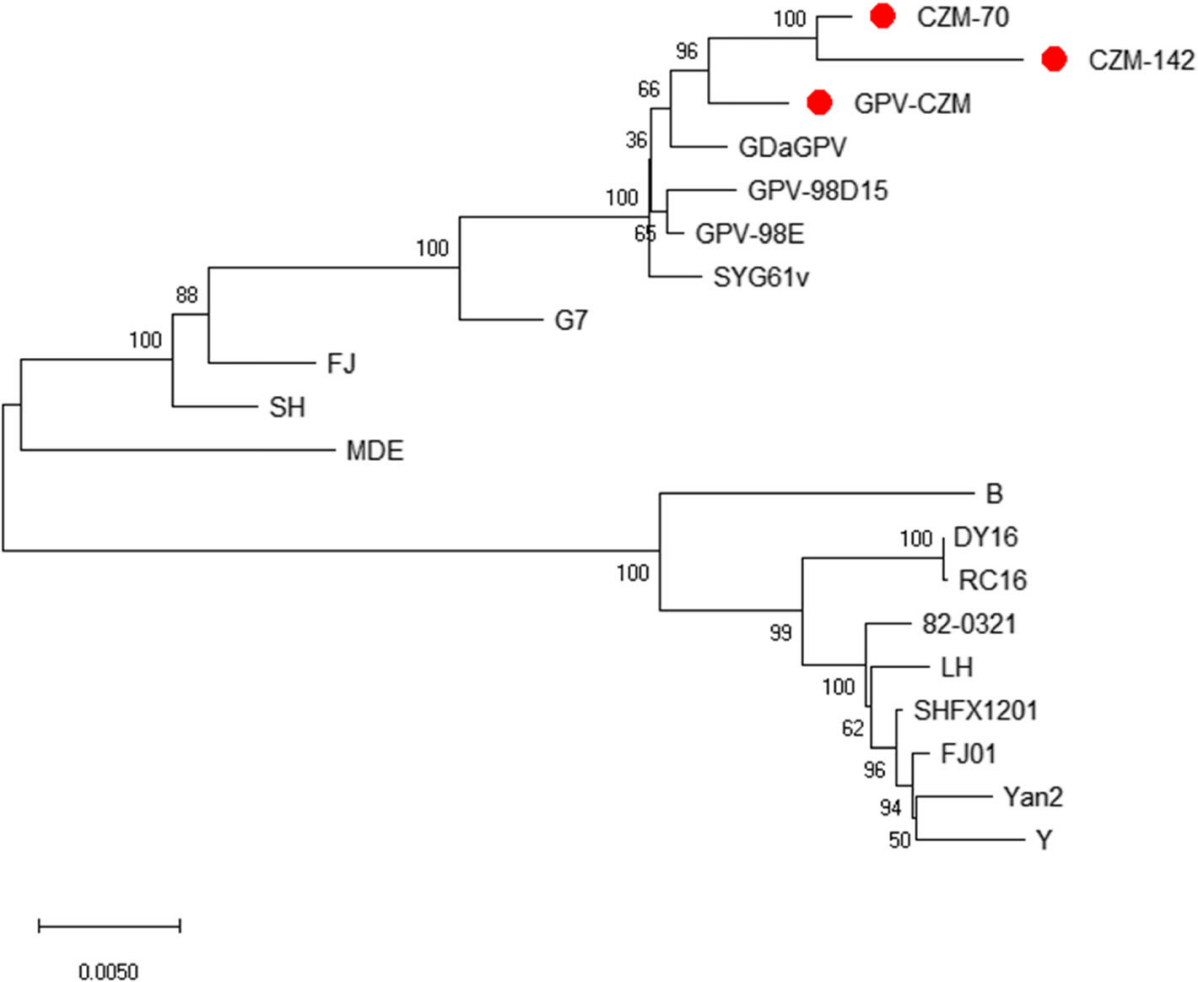
A phylogenetic tree for the genome of GPV-CZM, GPV-CZM-70, and GPV-CZM-142 was constructed using the neighbor-joining method (1000 bootstraps) with MEGA6

To profile sequence mutations in the genome of the GEF adapted GPV-CZM strains, the genomes of the GPV-CZM, GPV-CZM-70, and GPV-CZM-142 strains were amplified using primers listed in Table 2. As shown in Fig. 1, six fragments with the expected sizes were efficiently amplified. These amplicons were cloned into the pGEM®-T Easy vector, followed by sequencing the recombinant plasmids. The whole-genome sizes of GPV-CZM, GPV-CZM-70, and GPV-CZM-142 were 5106 bp, 5120 bp, and 5128 bp, respectively. The sequence of GPV-CZM, GPV-CZM-70, and GPV-CZM-142 had been submitted to the GenBank (the accession number for GPV-CZM, GPV-CZM-70, and GPV-CZM-142 was MT939902, MT939903, and MT939904, respectively). Alignment analysis of the genomes of the adapted GPV-CZM-70 and GPV-CZM-142 against the wild type GPV-CZM identified several adapted sites in the ITR region. Moreover, 60 single nucleotide mutations in the genome of GPV-CZM-142 compared with the genome of GPV-CZM. Among these mutations, 13, 16, and 31 sites were located in the non-coding region, the NS gene, and the VP gene. Genome phylogenic analysis indicated that GPV-CZM, GPV-CZM-70, and GPV-CZM-142 strains were clustered into the same branch as the strains GDaGPV, SYG61v, GPV-98E, and GPV-98D15 (Fig. 2). It is noteworthy that GPV-98D15 is an attenuated GPV through passaging in the duck embryo. The GPV reference strains used for phylogenic analysis were listed in Table 3.

**Table 2:** Primers used for GPV DNA amplification

Primer name	Primer sequence (5′-3′)	Position of the fragment
F1	TTCAGCTGCTCATTGGAGGGTT	1–203 (4903–5106)
R1	TTCTCGAGGCGTGGTCAACCTAACA	
F2	CGCATGCCGCGCGGTCAGCCCAAT	196–1013
R2	TATGTATGCTGCAGTCACGGTCTT	
F3	TAAGACCGTGACTGCAGCATAC	1009–2051
R3	TTCGCTCGTTCAGGAACGGGCTCTG	
F4	CAGAGCCCGTTCCTGAACGAG	2022–3041
R4	CAGAGCCCGTTCCTGAACGAG	
F5	CGAACCTGTGGCAGCATCTGAAATG	3010–4099
R5	GTCACTTATTCCTGCTGTAGTGCTG	
F6	CAGCACTACAGCAGGAATAAG	4072–4911
R6	CGCATGCGCGCGGTCAGCCCAATAG

**Table 3:** The information for 17 GPV reference strains used in this study

Strain	Genome/bp	Source	Login ID
GPV-98E	5106	Heilongjiang Province, China	KT598506
GPV-98D15	5114	Heilongjiang Province, China	KT598505
SYG61v	5102	Jiangsu Province, China	KC996729
DY16	5046	Jiangsu Province, China	MH209633
LH	5047	Jiangsu Province, China	KM272560
SH	5106	Shanghai, China	JF333590
SHFX1201	5050	Shanghai Province, China	KC478066
Y	5106	Anhui Province, China	KC178571
Yan2	5106	Anhui Province, China	KR136258
GDaGPV	5106	Fujian Province, China	HQ891825
G7	5106	Fujian Province, China	KR029617
FJ	5049	Fujian Province, China	KY511292
FJ01	5104	Fujian Province, China	KT232256
MDE	5106	Fujian Province, China	MF438102
RC16	5046	Sichuan Province, China	KY475562
82–0321	5050	Taiwan Province, China	EU583390
B	5106	Hungary	U25749

### Mutations identified in ITR of GPV-CZM-142

**Fig. 3:**
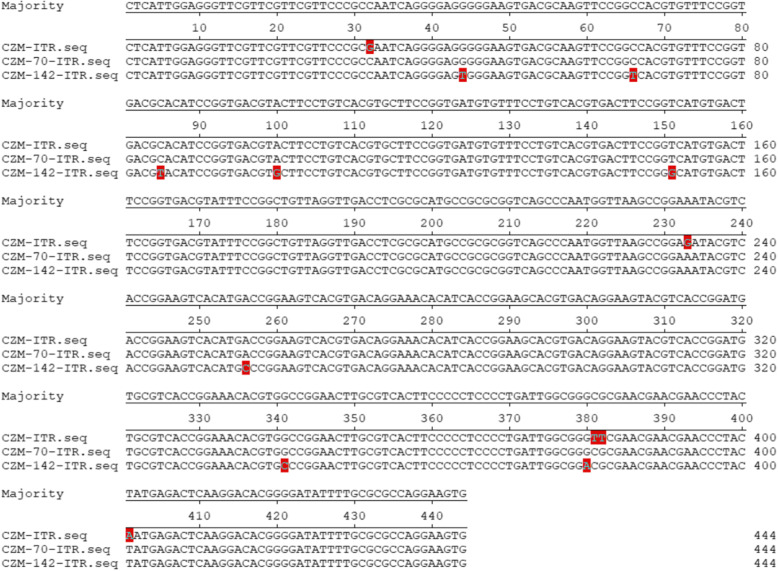
Adapted sites in the ITR region of GPV-CZM-70 and GPV-CZM-142. The ITR region of GPV-CZM, GPV-CZM-70, and GPV-CZM-142 was aligned, and several adapted sites were identified in ITR of GPV-CZM-70 and GPV-CZM-142, respectively, compared with that of the wild type GPV-CZM

Since ITR plays vital roles in gene expression and viral replication, ITR of the adapted GPV-CZM-70 and GPV-CZM-142 were further analyzed. Although no deletion or insertion was found in the ITR of GPV-CZM-70 and GPV-CZM-142 in comparison with that of the wild type GPV-CZM, the homology of these ITRs from GPV-CZM, GPV-CZM-70, and GPV-CZM-142 was 97.1–98.9%. As shown in Fig. 3, mutations G32C, G233A, T381C, T382G and A401T were found in the ITR of GPV-CZM-70, and mutations G32C, G44T, C66T, C85T, A100G, T151G, G233A, A256C, G341C, G380A, T381C, T382G and A401T were found in the ITR of GPV-CZM-70 when compared with the ITR of the wild type GPV-CZM.

### Mutations identified in NS and VP1 of GPV-CZM-142

Although no deletion and insertion were identified in the non-structural protein NS and the structural protein VP1 of GPV-CZM-70 and GPV-CZM-142 compared with the wild type strain of GPV-CZM, some single-nucleotide mutations were identified in the protein-encoding genes for both two strains (Tables 4 and 5). Compared with GPV-CZM, GPV-CZM-70 had 18 mutations for an amino acid with 2 mutations located in the NS gene (Table 4) and the other mutations located in the VP1 gene (Table 5). In contrast, GPV-CZM-142 carried 33 mutations for an amino acid with 8 mutations located in the NS gene (Table 4) and the other mutations located in the VP1 gene (Table 5). Notably, of these mutations, 7 and 16 mutations in NS and VP1 were common to GPV-CZM-70 and GPV-CZM-142, respectively. Besides, 7 and 6 mutations in NS and VP1 of GPV-CZM-142 were also found in GPV strain SYG61v, an attenuated vaccine strain. These findings suggest that these mutations are involved in host adaptation of the virus.

**Table 4:** Mutations in NS protein of GPV-CZM-70 and GPV-CZM-142

Position	GPV-CZM	CZM-70	CZM-142	SYG61v
72	I	V	V	I
149	Q	Q	L	Q
199	E	E	D	E
203	A	A	S	A
501	L	L	P	P
507	R	R	P	P
508	K	E	E	E
573	E	E	A	E
579	T	T	T	E

**Table 5:** Mutations in VP1 protein of GPV-CZM-70 and GPV-CZM-142

Position	GPV-CZM	CZM-70	CZM-142	SYG61v
3	T	N	N	T
5	L	V	V	L
89	Q	Q	R	Q
110	D	D	N	D
142	D	N	N	D
146	T	K	K	T
193	T	P	P	P
201	K	K	K	E
217	V	L	L	V
302	I	I	L	I
360	P	A	A	P
381	N	N	K	N
449	S	R	R	S
503	F	V	V	V
516	L	P	P	P
532	K	Q	Q	Q
534	I	M	M	I
537	I	L	L	L
544	S	P	P	S
545	G	G	S	G
546	S	S	T	S
547	T	T	S	T
549	A	A	T	A
555	R	M	M	M
593	D	E	E	D
643	V	V	I	V

## Discussion

The live attenuated GPV vaccine plays a critical role in preventing the disease caused by GPV. However, the frequent emergence of the GPV mutants in the vaccinated goose in China is challenging the current GPV controlling strategies, and the molecular basis for the attenuation of the highly pathogenic GPV needs to be elucidated. In this study, a highly attenuated GPV strain, GPV-CZM-142, was generated by blind passing a highly pathogenic strain GPV-CZM in GEFs. According to the viral titers of TCID_50_ and ELD_50_, the virulence of the host-adapted GPV-CZM-142 in GEF was more than 10,000 times weaker than that of the wild type GPV-CZM. Sequencing analysis revealed adapted mutations through the genome, however, no deletion and insertion were found in genome of GPV-CZM-70 and GPV-CZM-142. Notably, 13 mutations detected in the ITR of GPV-CZM-142 covered all the five mutations found in ITR of GPV-CZM-70, indicating the host adaptation of these five mutations in GPV-CZM-70. The previous study indicates that the deletion in the ITR may play roles in the attenuation of the GPV through host adaptation, and GPV strains with short ITR are thought to be less pathogenic [5]. Recently, Zhang et al. also report several insertions in the ITR of the attenuated GPV strains through adaptation in GEF and DEF compared with the wild type strain [6]. However, the roles of these deletions, insertions or, mutations identified here in the attenuation of GPV, need to be further investigated.

Although no insertion or deletion was found in the coding regions in GPV-CZM-70 and GPV-CZM-142 compared with the wild type strain, several mutations were identified in the NS, and VP1 protein-encoding genes in the two adapted GPV strains. As a non-structural protein, NS plays a vital role in initiating the viral replication and inducing apoptosis in the infected cells [7–9]. VP1, one of the major structural proteins, is thought to bind the cell receptor to mediate the infection of GPV and play critical roles in the pathogenesis of GPV. Recently, a rescuing assay of the recombinant GPV with different VP1 genes by Wang et al. indicates that the chimeric virus with VP1 derived from the virulent strain was pathogenic to goslings, whereas that with VP1 derived from the attenuated strain was non-pathogenic to goslings [10]. Liu et al. also report that 164 K, 165 K, and 167 K of VP1 were vital for the proliferation of GPV in vitro [11]. It is noteworthy that 7 mutations in NS and 16 mutations in VP1 were common to GPV-CZM-70 and GPV-CZM-142 compared with GPV-CZM. Interestingly, 7 mutations in NS and 6 mutations in VP1 of GPV-CZM-142 are also found in an attenuated vaccine strain, SYG61v. All these findings suggest that the mutations may contribute to the attenuation of the adapted GPV strains. Moreover, several mutations, such as K532Q, I534M, I537L, R555M, and D594E, in the VP1 found in GPV-CZM-70 and GPV-CZM-142 were located in or very close to the cell receptor binding domain of VP1, implying their host adaptation and potential roles in the attenuation of GPV. Notably, compared with the GPV-CZM-70, GPV-CZM-142 had 6 mutations (Q149L, E199D, A203S, L501P, R507P, E573A) in NS, 9 mutations in VP1 (Q89R, D110N, I302L, N381K, G545S, S546T, T547S, A549T, V643I) and large insertion in ITR. These additional mutations in NS and VP1 and the large insertion in ITR in the GPV-CZM-142 may contribute to the difference in virulence between GPV-CZM-70 and GPV-CZM-142.

## Conclusion

In summary, a novel highly attenuated GPV strain GPV-CZM-142 was generated through serial passages of the virulent strain GPV-CZM in GEF, and novel mutations related to the adaptation were identified in the genome of the GPV-CZM-142. The mutations in ITR affecting the activity of the ITR and contributing to the attenuated phenotype of the two adapted strains need to be confirmed by using the mini-genome system or by corresponding recombinant viruses.

## Methods

### Virus strains and goose embryos

A highly pathogenic GPV strain, GPV-CZM, was kept in our laboratory. From 11-day-old goose embryos without GPV maternal antibody, where the goose embryonic fibroblasts (GEFs) were isolated, were kindly provided by Dr. Zhang (Sinopharm Yangzhou VAC Biological Engineering Co. Ltd).

### Preparation of GEFs

Several 11-day-old goose embryos were selected for GEF preparation. The heads, limbs, and internal organs were first removed. The remaining embryo bodies were then rinsed with PBS, followed by digestion with trypsin. After filtering with 6 layers of gauze, the GEFs were cultured in DMEM (Gibco, NY, USA) supplemented with 10% FBS (fetal bovine serum) (Lonsera, Shanghai, China) at 37 °C with 5% CO_2._

### Virus passaging

The GEFs prepared above were infected with the GPV-CZM strain (1,10 dilution in PBS) for 2 h. After rinsed with PBS once, the infected GEFs were cultured in DMEM supplemented with 1% FBS for 5–7 d, followed by collection. The cells were then frozen and thawed once. The supernatants were isolated from the infected cells, followed by infecting newly isolated GEFs with it. Like this, the passaging of the virus went into the next cycle.

### TCID_50_ assay

GEFs were infected with the viruses that were diluted with DMEM containing 1% FBS at 1:10, 1:102, 1:103, 1:104, 1:105, and 1:106 in 96-well plates (6 wells per dilution). At day 5 post-inoculation, the cells were fixed by the ice-cold acetone: ethanol (3,2) mixture for 5 min and washed with PBS. The cells were then stained with the diluted monoclonal primary antibody specific to GPV for 45 min at 37 °C. After washing three times with PBS, the cells were stained with the diluted secondary antibody (goat anti-mouse IgG-FITC) for another 45 min at 37 °C. Again, after three washes with PBS, the cells were observed under inverted fluorescence microscopy. The TCID_50_ of these viruses was calculated by the Reed-Muench method based on indirect immunofluorescence intensity.

### ELD_50_ assay

Different GPV strains were diluted with PBS at 1:10, 1: 10^2^, 1: 10^3^, 1: 10^4,^ and 1: 10^5^ and injected into the allantoic cavity of 12 day-old goose embryos (5 embryos for each dilution). The morbidity and mortality of the inoculated goose embryos were recorded daily.

### Genome sequencing analysis

The viral DNA was isolated from different GPV strains using the DNA extraction kit and store at − 20 °C. The PCR primers based on the genome sequence of the standard strain B (U25749) were used (Table 1) to amplify the whole genome of GPV [5, 12]. The PCR products were then cloned into the pGEM®-T vector (Promega) and transformed into DH5α *E. coli* competent cells. The recombinant plasmids were extracted and sequenced by Huada Gene Technology Co., Ltd.

### Identification of sequence mutations

The whole-genome sequences from different GPV-CZM strains were aligned and compared using DNAStar Lasergene software. The regions of ITR, NS, and VP1 from two replicates of GPV-CZM were further analyzed with GPV reference strains using MEGA6.1 software (Table 1).

## Data Availability

The datasets used and/or analyzed during the current study are available from the corresponding author on reasonable request.

## Abbreviations

GPV: Goose parvovirus
GEF: Goose embryonic fibroblasts
ITR: Inverted terminal repeat
ORF: Open reading frame
NS: Non-structural protein
VP: Capsid protein
DMEM: Dulbecco’s modified Eagle’s medium
FBS: Fetal bovine serum
ELD_50_: 50% Embryo Lethal Dose
TCID_50_: 50% Tissue Culture Infectious Dose
